# The effects of exercise training in a weight loss lifestyle intervention on asthma control, quality of life and psychosocial symptoms in adult obese asthmatics: protocol of a randomized controlled trial

**DOI:** 10.1186/s12890-015-0111-2

**Published:** 2015-10-21

**Authors:** Patricia D. Freitas, Palmira G. Ferreira, Analuci da Silva, Sonia Trecco, Rafael Stelmach, Alberto Cukier, Regina Carvalho-Pinto, João Marcos Salge, Frederico LA Fernandes, Marcio C. Mancini, Milton A. Martins, Celso RF Carvalho

**Affiliations:** Department of Physical Therapy, University of São Paulo, Av. Dr. Arnaldo 455, Rm 1210, São Paulo, SP 01246-903 Brazil; Department of Psychology, University of São Paulo, Sao Paulo, Brazil; Department of Nutrition, University of São Paulo, Sao Paulo, Brazil; Department of Pulmonary Heart Institute (InCor), University of São Paulo, Sao Paulo, Brazil; Department of Medicine, School of medicine, University of São Paulo, Brazil, Av. Dr. Arnaldo 455 – room 1210, Sao Paulo, SP 01246-903 Brazil

**Keywords:** Asthma, Obesity, Weight loss, Exercise training, Asthma control

## Abstract

**Background:**

Asthma and obesity are public health problems with increasing prevalence worldwide. Clinical and epidemiologic studies have demonstrated that obese asthmatics have worse clinical control and health related quality of life (HRQL) despite an optimized medical treatment. Bariatric surgery is successful to weight-loss and improves asthma control; however, the benefits of nonsurgical interventions remain unknown.

**Methods/Design:**

This is a randomized controlled trial with 2-arms parallel. Fifty-five moderate or severe asthmatics with grade II obesity (BMI ≥ 35 kg/m^2^) under optimized medication will be randomly assigned into either weight-loss program + sham (WL + S group) or weight-loss program + exercise (WL + E group). The weight loss program will be the same for both groups including nutrition and psychological therapies (every 15 days, total of 6 sessions, 60 min each). Exercise program will include aerobic and resistance muscle training while sham treatment will include a breathing and stretching program (both programs twice a week, 3 months, 60 min each session). The primary outcome variable will be asthma clinical control. Secondary outcomes include HRQL, levels of depression and anxiety, lung function, daily life physical activity, body composition, maximal aerobic capacity, strength muscle and sleep disorders. Potential mechanism (changes in lung mechanical and airway/systemic inflammation) will also be examined to explain the benefits in both groups.

**Discussion:**

This study will bring a significant contribution to the literature evaluating the effects of exercise conditioning in a weight loss intervention in obese asthmatics as well as will evaluate possible involved mechanisms.

**Trial registration:**

NCT02188940

## Background

Asthma is a chronic respiratory disease characterized by episodes of reversible airway obstruction, chronic airway inflammation and airway hyperresponsiveness. Its symptoms include wheezing, shortness of breath, chest tightness and cough, which may resolve either spontaneously or with the administration of an appropriate treatment [1]. Asthma affects approximately 300 million people worldwide and is expected to affect an additional 100 million people by 2025 [1, 2]. The dramatic increase in the prevalence of asthma over the past few decades has occurred in conjunction with an increase in the prevalence of obesity [3, 4], a finding suggestive of a possible relationship between the two conditions. Obesity is characterized by excess fat accumulation in the body as a result of a complex interaction among genetics, dietary caloric intake and energy expenditure and is often diagnosed in adults with a BMI ≥ 30.0 kg/m^2^ [5]. Obesity also affects a large number of individuals, as more than 200 million men and nearly 300 million women are obese. The prevalence of obesity is highest in WHO Regions of America (27 % of obesity) and lowest in the WHO Regions for South East Asia (5 % of obesity) [6].

Many cross-sectional epidemiologic studies have demonstrated a relationship between asthma and obesity [7–9]. Obesity is a major risk factor for asthma, as overweight and obese subjects are 38 % and 92 % more likely to develop asthma, respectively, than patients with normal weights [10]. Additionally, obesity results in the development of a difficult-to-control asthma phenotype in which patients experience worse clinical control, poorer quality of life, reduced lung function, poor responses to corticosteroids and more psychosocial symptoms [11–14]. As obesity is a risk factor for the development of asthma, Global Initiative for asthma (GINA) 2014 [1] recommends weight loss for all obese asthmatics.

Some interventions have been considered for weight loss in obese asthmatic patients and bariatric surgery has been suggested as a means of improving asthma control, lung function and decreased medication usage [15, 16]. However, the most recent guidelines pertaining to the management of obesity recommend utilizing a comprehensive approach to weight-loss intervention focused on diet, physical activity and behaviour self-management as a first-line therapy [17].

A recent Cochrane review found only 4 randomized controlled trials (RCTs) pertaining to weight loss in the setting of asthma [18] via nonsurgical intervention. Two trials used meal replacement with a very low calorie diet [19, 20], and Dias-Junior et al. [21] combined a low calorie diet with anti-obesity drugs (orlistat and subtramine). Each of these studies observed positive effects following weight loss. The study by Scott et al. [22] was the only to include physical activity as an adjuvant treatment; the authors observed that the patients who participated in physical activity did not experience additional benefits in terms of weight loss and improvements in clinical control, although this finding may have overlooked the levels of physical activity in which these patients participated at baseline. Therefore, the Cochrane review study did not find any studies using a comprehensive approach to lifestyle intervention focused on diet, physical activity and behaviour self-management as recommended by the most recently published guidelines [17].

Recent evidence suggests that improving physical fitness among patients with asthma is important because it improves airway hyperreactivity [23], psychosocial factors [24], and health related quality of life [25], and also reduces the need for corticoid administration [23, 26]. Moreover, recent studies have also demonstrated the anti-inflammatory effects of exercise training in patients with asthma [27, 28]; these results are consistent with those of studies utilizing animal asthma models [29–31]. Additionally, improvements in physical fitness also effect the immune system in obese subjects by decreasing the activity of pro-inflammatory mediators [Interleukin (IL-6), monocyte chemotactic protein-1 (MCP-1), C-reactive protein (CRP), IL-8 and tumour necrosis factor (TNF-α)] and increasing the levels of anti-inflammatory markers and mediators (adiponectin and IL-10) [32–34]. Therefore, exercise not only plays a role in lifestyle interventions intended to facilitate weight loss in asthma but is also an intervention in and of itself.

As previously discussed, asthma and obesity are clinical conditions characterized by chronic inflammation; the effects of exercise have been evaluated in the setting of each condition but not in the setting of both diseases together. Our hypothesis is that exercise training amplifies weight loss and exerts both anti-inflammatory and immunoregulatory effects, resulting in improved clinical control and quality of life, as well as improved psychosocial symptoms and sleep disorders among obese asthmatic patients. The objective is to assess the effects of exercise training in a weight-loss program on asthma control, quality of life and psychosocial symptoms in obese patients with stable asthma.

## Methods

### Study design

**Fig. 1:**
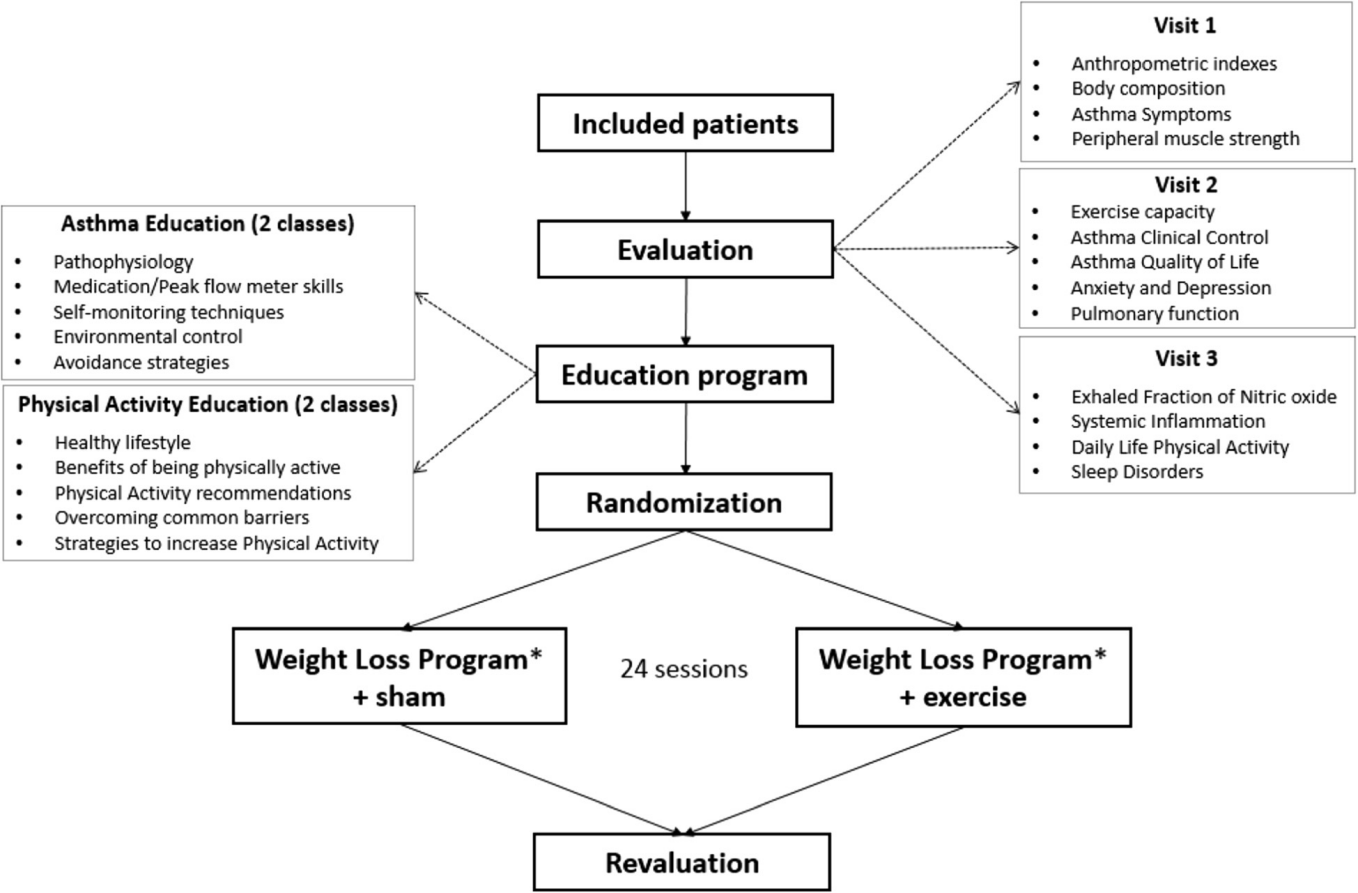
Study flow chart. After being included in the study, the patients will be evaluated during 3 non-consecutives visits. Each of the groups will receive the same education program and will participate in the same weight loss program* (composed of nutrition and psychological therapy); however, only one of the groups will participate in exercise training, as the remaining group will perform a sham intervention (breathing and stretching exercises). The groups will performed 24 intervention session, after which they will undergo a second evaluation utilizing the same tests as the baseline evaluation

This is a prospective and randomized open-label controlled trial with 2 arms and blinded assessments. Both groups will receive similar educational and weight loss interventions (composed of both nutritional and psychological therapies), but only one group will perform exercise training. The study design is depicted in Fig. 1.

### Study setting

The patients will be recruited from an outpatient asthma clinic at a University hospital. The Hospital Research Ethics Committee of the University of Sao Paulo approved the study, (07137512.9.0000.0068) and all patients provided written informed consent before participating. This study is registered on ClinicalTrials.gov as NCT02188940.

### Eligibility criteria

Patients between 30 and 60 years of age with class II obesity (BMI ≥ 35 kg/m^2^) and either moderate or severe persistent asthma according to the GINA criteria [1] will be eligible to participate. The subjects will have to be under medical treatment for at least 6 months and to have clinically stable disease (no hospitalizations, emergency care or medication changes for at least 30 days). The patients should will also be physically inactive as defined by the American College of Sports Medicine’s (ACSM) guidelines (less than 60 min of structured or planned physical activity per week) within the last 6 months [35]. The exclusion criteria included the following: the presence of another pulmonary disease, either cardiovascular or musculoskeletal diseases that may interfere with the patient’s evaluations or impair exercise training, active cancer, a weight change greater than 5 % within the last 6 months, taking anti-obesity drugs within the past 3 months, a history of bariatric surgery, either uncontrolled hypertension or diabetes, and the use of continuous positive airway pressure. Patients who are participating in another research protocol or are unable to understand our questionnaire, as well as smokers or ex-smokers (≥10 pack-years), pregnant women or breast feeding women, will also be excluded. The interventions will be performed between 2 medical appointments in order to avoid medication changes.

### Experimental design

The patients will be assessed before and after the interventions on 3 non-consecutive days. On day 1, anthropometric indexes, body composition, asthma symptoms and peripheral muscle strength will be assessed. On day 2, asthma clinical control will be assessed via an asthma control questionnaire (ACQ), an asthma quality of life questionnaire (AQLQ), and a hospital anxiety depression scale (HADS); sleep disorder history, lung function and exercise capacity will be also assessed. On day 3, the exhaled fraction of nitric oxide (FeNO), daily life physical activity and systemic inflammation will be measured. Following these baseline evaluations, all eligible patients will receive a 6-h educational program and will be subsequently randomly assigned into the following 2 groups: either a weight loss program (including nutritional and psychological therapy) with a sham treatment (WL + S group), or the same weight loss program with exercise (WL + E group). All patients will complete 24 treatment sessions, after which they will be reevaluated.

### Allocation, randomization and blinding

The eligible patients will be randomly allocated to their respective intervention group. The randomization schedule will be computer-generated and carried out by an investigator blinded to the recruitment, evaluation and treatment of the participants. Each patient’s allocation will be concealed using sequentially numbered, sealed and placed in opaque envelopes. The researcher that will provide the treatments who will be not involved in the data collection, will open the envelopes and will inform the group allocated to each participant. The participants will be informed if they would receive 1 out 2 different interventions to facilitate weight loss. Due to the nature of the interventions, it will not be possible to blind the physiotherapist that will provide the exercise training and the breathing and stretching programs; however, the nutritional and psychological interventions, as well as the assessments of each of the variables and the data analysis, will be given in a blinded manner.

### Interventions

#### Educational program

Both groups will complete an educational program consisting of 4 classes held twice a week before the interventions, each lasting 90 min. The 2 first classes will be based on an education videotape, the *ABC* of asthma, as well as presentations and group discussions pertaining to asthma pathophysiology, medication and peak flow meter skills, self-monitoring techniques, environmental control and avoidance strategies in accordance with previous studies [24, 36] and asthma guidelines [1]. During the 2 last classes, the patients will also receive information and educational materials regarding current international physical activity recommendations and the benefits thereof [35].

#### Nutritional intervention

A nutritionist will conduct the nutritional program with visits every 2 weeks during the 3 month intervention period, for a total of 6 sessions of 60 min each for both groups. The patients will be advised to follow a hypocaloric diet in which the calorie intake of each patient will be estimated by multiplying adjusted body weight (at a BMI of 25 kg/m^2^) by 20 calories [37]. Food consumption will be evaluated using a 24-h food record, and all caloric intake, macronutrients and micronutrients will be quantified using NutWin® software (recommended dietary allowances based on a food pyramid) [38]. The patients will complete the food dairy at the beginning (1^st^ session), the middle (3^rd^ session) and the end (6^th^ session) of the sessions. During each session, the nutritionist will measure and record the patients’ weights, discuss the importance of maintaining a balanced diet, and promote appropriate daily food intake and make each patient aware of the responsibility one bears in controlling one’s weight (Table 1).

**Table 1:** Nutritional program

No. session	Activities
1	Presentation of the group and the nutritional program
	The measurement and recording of body weights, concepts of BMI/obesity range
	A balanced food pyramid and how to complete the 24 hour food intake dairy
2	The measurement and recording of body weight
	Individualized calorie goals with dietary suggestions
	Daily food routine and portion sizes
3	The measurement and recording of body weight
	The role of carbohydrates, proteins and fats in a diet
	Choices of low-energy and nutrient-dense meals and snacks
4	The measurement and recording of body weight
	The role of vitamins and minerals
	The ideal amount of nutrient intake to lose weight and stay healthy
5	The measurement and recording of body weight
	Healthy food preparation techniques and proposals for healthy meals
	The risk of diabetes and hypertension associated with obesity (comorbidities)
6	The measurement and recording of body weight
	“Virtual restaurant” and how to read a food nutrition labels
	Careful selection of restaurants and the finalization of the nutrition program

*BMI* body mass index, *No.* number of session. The nutritional program will be performed every 15 days over 3 months for a total of 6 sessions of 60 min each

#### Behavioural therapy

A psychologist will provide the therapy every 2 weeks for a total of 6 sessions of 60 min each for both groups. Group sessions using behavioural techniques (such as self-management, motivational strategies, positive reinforcement and relapse prevention) will be provided in order to improve patient adherence to the weight loss program [39]. The interventions will be based on the Transtheoretical model, which recognizes that behaviour changes are dynamic processes that move through stages and reinforces change via goal setting, skill development and self-control [39]. The psychologist will discuss several issues related to behaviour changes, using relaxation techniques, internal experiences and patient self-reporting (Table 2). The nutritional and psychological programs will be similar for both groups but they will be performed separately for each group to avoid that patients from distinctive groups meet themselves.

**Table 2:** Psychological program

No. session	Activities
1	Presentation of the participants
	Explanation of the psychological program
	Assessment of patients’ expectations regarding weight loss
2	Breathing relaxation techniques to control anxiety
	Experiences concerning weight loss (successes and relapses)
	Identification of the reasons for and the onset of obesity in each patient
3	Emotional versus physiological hunger (learning to listen to the body)
	Feeding behaviour and pleasure from food
	The identification of personal barriers to weight loss and potential solutions to overcoming these barriers
4	Self-efficacy (dynamic group using drawings)
	A review of self-monitoring records and progress
	Realistic goal setting and action plans
5	Dynamic group called “time for self-care”
	Responsibility regarding food choices
	How to deal with relapses during the program
6	Dynamic group activities using the mirror to increase each patient’s morale
	A review of self-monitoring records and progress
	Finalization of the program

*No.* number of session. The psychological program will be completed every 15 days over 3 months for a total of 6 sessions of 60 min each

#### Exercise training program

The exercise training program will include aerobic and resistance exercises supervised by a physiotherapist and will be offered to only one of the groups (WL + E group). The program will be performed twice a week for 3 months for a total of 24 sessions of 60 min each. In order to avoid joint discomfort and improve patient adherence to the exercise program, aerobic training will be performed on both a treadmill (Jog 700, Technogym, Italy) and either a bike (Bike 700, Technogym, Italy) or an elliptical (Syncro 700, Technogym, Italy) machine. The intensity of the aerobic training will be based on peak oxygen consumption (peak VO_2_) and monitored using each patient’s target heart rate (THR). Exercise intensity will begin with a THR of 50 to 60 % of peak VO_2_ and will be increased by 5 % every 2 weeks based on symptoms and perceived exertion, using the modified Borg scales for leg discomfort and dyspnea [40], reaching a maximum of 75 % of peak VO_2_ [41]. Each patient will receive an accelerometer in order to record daily numbers of steps during the 1^st^, 6^th^ and 12^th^ weeks to encourage an increased physical activity level. In order to complete the amount of weekly physical activity recommended by the international guidelines [35], the patients will be also advised to walk at least twice a week for 30 min and to complete a daily physical activity record. Both heart rate and the modified Borg scale [40] will be assessed during each session before exercise, every 10 min during exercise and after exercise. Peak expiratory flow (PEF) will be measured before each session, and inhaled salbutamol (200 μg) will be recommended if values < 70 % were observed. The safety of the exercise training will be monitored by quantifying PEF, blood pressure and asthma symptoms before and after each exercise session as previously described [28].

The aerobic training will be interspersed with resistance training for the upper and lower limbs, targeting the following major muscle groups: chest, deltoid, quadriceps and hamstrings. Patients will begin the resistance training by performing 2 sets of 10 repetitions with an intensity of 50 % to 70 % of the one-maximal resistance test (1-RM) [42]; progression will take place first in the number of repetitions (until 2 sets of 15 repetitions are reached). When each patient reaches 15 repetitions, the load will be increased from 1 to 3 kg for the exercises for the upper limbs and 5 to 10 kg for the lower limbs, depending on the muscle being exercised and the patient’s tolerance level.

#### Sham treatment

The breathing and stretching exercise programs will be completed only by the WL + S group; the sessions will be performed twice a week for 12 weeks and will be supervised by a physiotherapist. The breathing and exercise programs will be performed as a sham treatment as previously described [24, 28], without an intensity progression. The program will be based on Yoga’s pranayama breathing exercises, including *kapalabhati*, *uddhiyana* and *agnisara,* as previously described [43]. Briefly, every exercise will be completed in 3 sets with 2 min of exercise intercalated with 60 s of rest for a total of 30 min each session. The stretching program will consist of exercises for the following major muscle groups: trapezius, pectoralis, gluteus, hamstrings, quadriceps femur, paraspinal, latissimus dorsi and pubic adductors. The exercises will be completed in 2 sets of 10 s each for a total of 30 min each session. The safety of these exercises will be monitored in the same way as the exercise training. The breathing and stretching exercises will be included as a sham intervention in the WL + S group in order to minimize the differences in the numbers of hospital visits and the amounts of patient attention between the two groups, not to induce respiratory breathing training benefits.

### Outcomes

#### Primary outcome

The primary outcome of this study will be the absolute change in the asthma control questionnaire (ACQ) post intervention between the groups following the intervention. The ACQ is both a reliable and validated method of measuring the impairment in asthma control [44, 45] and includes specific parameters regarding both daytime and nocturnal asthma symptoms, activity limitations, dyspnea, wheezing and rescue bronchodilator use within the last week (a short-acting B_2_-agonist) [45]. An additional question assessing forced expiratory volume in 1 s (FEV_1_) (% predicted, pre-bronchodilator) is completed by the researcher. The ACQ contains 7 items rated on a 7-point scale (0 = without limitation, 6 = maximum limitation), with a higher score indicating worse control. The ACQ has been validated for Brazilian Portuguese [46]. Previous studies have demonstrated that scores lower than 0.75 points are associated with good asthma control, whereas scores greater than 1.5 points are indicative of poorly controlled asthma [44]. A change of at least 0.5 points on the ACQ is regarded as clinically significant [47].

### Secondary outcomes

#### The asthma quality of life questionnaire (AQLQ)

The asthma quality of life questionnaire [48, 49] consists of 32 items rated on a 7-point scale (1 = great deal, 7 = not at all) divided into the following 4 domains: activity limitations, symptoms, emotional function and environmental stimuli. The AQLQ has been translated and validated for Portuguese patients [50]. A higher AQLQ score indicates a better quality of life; clinically effective treatment resulted in a 0.5 point increase in the score following the intervention [51].

#### Levels of anxiety and depression

Symptoms of anxiety and depression will be evaluated using the Hospital Anxiety and Depression Scale (HADS) [52], which consists of 14 items divided into 2 subscales (7 for anxiety and 7 for depression). Each item is scored from 0 to 3, with a maximum score of 21 points for each subscale. A score greater than 9 in each subscale suggests a diagnosis of either anxiety and/or depression [53].

#### Asthma symptoms and exacerbations

Asthma symptoms and exacerbations will be evaluated using a symptom diary as previously reported [24]. The asthma diary includes questions about episodes and symptoms (coughing, wheezing, shortness of breath, nocturnal awakenings and the number of puffs of as-needed β_2_-agonist). Days free of asthma symptoms will be considered when the patient did not report any symptoms; these days will be totalled on a monthly basis. Asthma exacerbations will be defined as an increase in symptoms associated with at least one of the following criteria: the use of ≥ 4 puffs of rescue medication per 24 h during a 48-h period, a need of systemic corticosteroids, an unscheduled medical appointment, and either a visit to an emergency room or a hospitalization.

#### Sleep disorders

The Berlin questionnaire will be used to estimate the risk (low to high) of obstructive sleep apnoea syndrome (OSA) [54]. The questionnaire consists of 10 items, divided into the following 3 domains: snoring and witnessed apnoeas (5 items), daytime sleepiness (4 items) and high blood pressure/obesity (1 item). Each category is classified as positive if the score is ≥ 2 points. Additionally, if the patients have at least 2 positive categories, they will be considered high risk for developing OSA. The Berlin questionnaire has been translated and validated for Portuguese [55]. The ActiSleep monitor (Pensacola, FL, USA) will be also used to objectively evaluate the sleep of patients. The participants will be instructed to use the monitor over a period of 7 consecutive nights on their non-dominant wrist. During this monitoring period, the participants will keep a sleep diary in which they will record the times at which they fall asleep, as well as when they awake each morning. We will analyze the following parameters: the total amount of sleep, sleep latency, the number and duration of awakenings and sleep efficiency [56].

#### Daily life physical activity (DLPA)

The accelerometer “ActiGraph GT3X” (ActiGraph, Pensacola, FL, USA) will quantify objectively the absolute change in DLPA. This device uses a solid-state tri-axial accelerometer to collect motion data on 3 axes (vertical and horizontals right-left and front-back) and measures and records time-varying accelerations [57]. The counts obtained in a given time period are linearly related to the intensity of the physical activity monitored during this period [58]. All units will be initialized via a computer interface to collect data in 60-s epochs in the 3 axes using specific software (ActiLife 6.9.5 Firmware version). Each participant will be instructed to use the accelerometer over a period of 7 consecutive days, with the device positioned securely on the patient’s hip (non-dominant side) using an elastic belt. During the study period using the accelerometer, the patients will keep a diary in which they will record bathing times and the times at which they begin to fall asleep and awake each morning. The accelerometer will record automatically each patient’s energy expenditure (Kcal), metabolic equivalent unit (MET), step counts, sedentary behaviour, and cut off points, as well as time (%) when sedentary or engaged in light, moderate or vigorous activity, and time (%) in the standing, sitting and supine positions [59].

#### Body composition

Body composition will be analyzed via an octopolar tactile bioelectrical impedance analysis (InBody720 - Biospace, Seoul, South Korea). An electrode system separately measures the impedance of the subject’s trunk, arms, and legs at 6 different frequencies (at 1, 5, 50, 250, 500 and 1000 kHz) and evaluates body segment. The equipment will analyze the percentages of body fat mass, soft lean mass, fat free mass, skeletal muscle mass and visceral fat area (cm^2^). The equipment has a high test-pretest reliability and accuracy [60]. The patients will be advised to fast for at least 4 h, refrain from physical activity for 8 h prior to testing, and avoid taking diuretics at least 24 h before testing [61].

#### Anthropometric indexes

The patients’ heights, weights (Filizola®, Brazil), waist circumferences, hip circumferences and waist to hip ratios (WHRs) will be measured using a standardized protocol [62, 63]. BMIs will be obtained by dividing patients’ weights in kilograms by their heights in meters squared (kg/m^2^) [64].

#### Cardiopulmonary exercise test (CPET)

The cardiopulmonary exercise test will be performed using an electrical cycle ergometer (Corival, Lode B.V.; Medical Technology, The Netherlands) linked to a digital equipped with an exercise evaluation system (CardioO_2_ System; Medical Graphics Corporation), in accordance with American Thoracic Society/American College of chest physicians (ATS/ACCP) guidelines [65]. Oxygen saturation (SpO_2_), as measured via pulse oximetry (Onyx, model 9500; Nonin, Plymouth, MN), and electrocardiography (Welch Allyn CardioPerfect, Inc., NY) will be monitored continuously during the tests. The following variables will be recorded breath-by-breath during rest, during exercise and following testing: work rate (WR), VO_2_, minute ventilation (VE), carbon dioxide production (VCO_2_), respiratory exchange rate (RER) and heart rate (HR). Additionally, blood pressure, the Borg score for leg discomfort and dyspnea, and inspiratory capacity (IC) will be measured at rest and every 2 min during testing until the end of testing [40]. The patients will perform a ramp-symptom-limited CPET consisting of 2 min of rest, 2 min of warm-up (unloaded pedalling) and an incremental work period (an increase from 10 to 20 W/min, taking into account the patient’s level of daily activity [66]. The predicted CPET values will be obtained from the Brazilian population [67].

#### Peripheral muscle strength

The maximal strength tests will be evaluated via a one repetition maximum test (1-RM) test in order to determine the load used at the beginning of resistance training [42]. The 1-RM test is defined as the maximum weight that an individual can lift in a single repetition and will be performed as previously described [42]. Briefly, the participants (1) will be familiarized with the equipment using minimal resistance; (2) will perform a 3-min warm-up period; (3) will complete 8 repetitions at approximately 50 % of the estimated 1-RM, followed by 3 repetitions of approximately 70 % of the estimated 1-RM; and (4) will complete single repetitions (maximum 5 trials) utilizing progressively heavier weights in order to meet the 1-RM. The recovery period among series should not be less than 1 min or more than 5 min. The 1-RM test will involve the following movements: extension of the arms, inclined bench press and seated leg press and extension of the feet.

### Evaluating possible mechanisms

#### Pulmonary function

Spirometry and lung volume measurements will be performed using a calibrated whole-body plethysmograph (Medical Graphics Corporation - MGC, St Paul, Mm, USA) according to both American Thoracic Society and European Respiratory Society (ATS/ERS) recommendations [68, 69]. Data related to IC, total lung capacity (TLC), expiratory reserve volume (ERV), functional residual capacity (FRC) and residual volume (RV) will be expressed both as totals and as percentages of predicted values [70]. A spirometer will be used to measure FEV_1_, forced vital capacity (FVC), forced expiratory flow 25–75 (FEF 25-75 %), peak expiratory flow (PEF) and maximal voluntary ventilation (MVV). Spirometry evaluations will be conducted before and after the administration of 200 mcg of inhaled salbutamol. Increases of either 12 % or 200 ml in FEV_1_ will be categorized as positive responses to bronchodilator therapy [68]. The predicted values will be provided by Pereira et al. [71].

#### Airway inflammation

Airway inflammation will be quantified using the exhaled fraction of nitric oxide (FeNO), using a portable analyser (NIOX MINO®; Aerocrine AB, Solna, Sweden), in accordance with ATS/ERS guidelines [72]. The patients will be asked to exhale fully while seated before inhaling through a NIOX filter until they will reach their total lung capacity, and immediately will exhale at a constant flow rate of 50 ml/s using a visual feedback system. The average levels of at least 3 acceptable measurements will be used. The patients will be instructed to avoid eating foods containing nitrate and caffeine and to avoid smoking and exercise 24 h before testing, as well as to refrain from ingesting either food or water for at least 2 h before testing. NO collection will be performed by the same professional at the same time of day in order to avoid changes in patients’ circadian rhythms. A cut off point of 25 parts per billion (ppb) will be used to either confirm or exclude a diagnosis of eosinophilic airway inflammation [72].

#### Systemic inflammation

Patients’ inflammatory systemic profiles will be assessed using blood-based markers. Venous blood samples will be collected following at least 8 h of overnight fasting, and the patients will be advised to avoid exercise, alcoholic and caffeinated beverages 24 h before testing. The cytometric bead array method (BD Biosciences, San Jose, CA, USA) will be used to analyse the levels of IL-1, IL-2, IL-4, IL-5, IL-6, IL-8, IL-10, IL-12, IL-13, TNF-α, vascular endothelial growth factor (VEGF), T-cell receptor beta (TGF-β) and the chemokines MIG/CXCL9, IP-10/CXCL10, IL-8/CXCL8, MCP-1/CCL2 and RANTES/CCL5. Serum leptin, adiponectin, CRP (ELISA, MILLIPORE), cortisol (Siemens, Immulite 200) and vitamin D (Ria-CT) levels will also be analyzed.

### Data analysis

#### Sample size

Sample size was calculated based on the minimal important difference of 0.5 points between the groups [47] on the ACQ questionnaire, with a standard deviation of 0.72 [73]. A sample size of 55 patients will be sufficient to detect an effect size of 0.5 between the 2 arms for 80 % alpha 2-sided, assuming up to a 20 % loss to follow-up.

#### Statistical analysis

An intention-to-threat analysis will be used to preserve the effects of group allocation and provide an assessment of the practical impact of the treatment [74], as recommended by the CONSORT statement [75]. The normality of continuous outcomes will be assessed via the Kolmogorov-Smirnov test. Comparisons of the initial and final data will be analyzed via a two-way repeated-measures analysis of variance, and the categorical outcomes, via the *χ*^2^ test. A partial correlation will be used to test the effects of airway and systemic inflammation as mediators. P values ≤ 0.05 will be considered statistically significant. The statistical analysis will be blinded to the treatment allocation and will be performed using specific software (SigmaStat 3.5, Systat Software Inc.).

## Discussion

The association between asthma and obesity is an interesting issue since the pathogenesis of asthma is altered in obese asthmatics because they do not respond as well to standard controller asthma therapy. The inclusion of evidence-based weight loss strategies is recommended by the GINA guidelines in spite of the lack of trials demonstrating the intervention that is most effective in facilitating weight loss in obese asthmatics [18, 22].

The aim of the present study is to assess the effects of exercise training in the setting of a weight loss intervention on asthma control, quality of life and psychosocial symptoms in obese asthmatics. It is important to understand the role of exercise as part of a comprehensive weight loss program in these patients, as well as to investigate possible mechanisms by which improvements in physical fitness result in improvements in both clinical and psychological variables. Additionally, sedentary lifestyle and deconditioning both play key roles in the development of respiratory symptoms in obese asthmatic patients [76]. Furthermore, exercise is associated with improvements in airway hyperreactivity [23], psychosocial factors [24], health related quality of life [25] and reduced airway inflammation [28] among patients with asthma. Additionally, exercise exerts positive effects on body weight, cardiovascular disease risk factors and systemic inflammation among patients who are obese [32–34, 77].

Nevertheless, we are aware of only 2 studies investigating the effects of physical fitness among obese asthmatic [22, 78]. Scott et al. [22] conducted a randomized trial involving 46 overweight and obese adults with asthma who were allocated to 3 distinct groups as follows: a calorie-restricted diet group, an exercise training group and a group receiving both interventions. Contrary to our study hypothesis, the authors observed that exercise training did not provide benefits in terms of weight loss and improvements in clinical control on a calorie-restricted diet program. It is important to note that they did not observe improvements in physical activity levels following their exercise training program and did not perform evaluations pertaining to improvements in physical fitness [22]. A possible explanation for their findings is that their patients participated in a greater amount of physical activity at baseline (61 % reported participating in vigorous activity, and 24 % reported participating in moderate physical activity). Furthermore, they were already close to the 10.000 steps/day threshold, suggesting they were physically active with respect to international guidelines [79]. A recent trial [78] investigated the effects of an evidence-based comprehensive weight loss intervention composed of diet, physical activity and behavioural therapy compared with standard therapy in obese asthmatics. In spite of the inclusion of a comprehensive weight loss intervention, the physical activity program in this study included recommendation to maintain a minimum of 150 min of physical activity per week and not a supervised exercise protocol. The patients in the intervention group experienced significantly more weight loss compared with the control subjects (respectively, −4.0 ± 0.8 kg vs. -2.1 ± 0.8 kg); however, neither group observed any changes in either ACQ or the numbers of asthma exacerbations.

The possible mechanisms underlying the improvements in patients’ physical fitness may be related to changes in lung mechanics and either systemic or airway inflammation. We expect that the addition of exercise training may have clinical benefits (reduced symptoms) among obese asthmatic patients by decreasing asthma allergic inflammation [28], as well as by amplifying weight loss by increasing metabolic consumption, which may subsequently decrease the low grade inflammation commonly noted among patients who are obese [33, 34]. Weight loss exerts positive effects on lung function among subjects with and without asthma [80] via improvements in chest wall compliance secondary to reduced mass loading effects caused by fat accumulation in and around the chest wall. If our hypothesis is correct, reductions in body weight will improve ventilation efficiency by improving chest wall compliance.

Improvements in physical fitness may reduce the levels of pro-inflammatory mediators observed in subjects with low-grade inflammation (high-sensitivity C-reactive protein, IL-6, TNF-α, MCP-1, IL-8, and leptin) [81–83] and increase the levels of anti-inflammatory markers and mediators (adiponectin and IL-10) [32–34]. We expect that exercise training will facilitate decreases in the levels of the systemic biomarkers that contribute to the pathogenesis of asthma, as it has been demonstrated previously that aerobic conditioning improves airway inflammation via reductions in FeNO levels among patients with asthma [28]. It has also been suggested that exercise training modulates allergic inflammation by increasing the expression of the anti-inflammatory cytokines IL-10 and IL-1ra in an animal model of asthma [84]. We hypothesized that there is a correlation between reduced FeNO, a marker of airway inflammation, and increased levels of these anti-inflammatory cytokines. Another possible effect of improved physical fitness is an improved quality of sleep and a reduction in psychological symptoms, as the prevalence of anxiety and depression are significant in both patients with asthma [85] and patients who are obese [85–87].

In conclusion, this study will bring a significant contribution to the literature evaluating the effects of exercise conditioning in a weight loss program; the results may facilitate improvements in asthma control, quality of life and psychosocial symptoms and help elucidate the possible mechanisms underlying the improvements in lung function, as well as both airway and systemic inflammation, among obese asthmatic patients.

## Abbreviations

HRQL: Health related quality of life
BMI: Body mass index
WL + S: Weight-loss program + sham treatment group
WL + E: Weight-loss program + exercise group
RCTs: Randomized controlled trials
IL: Interleukin
MCP-1: Monocyte chemotactic protein-1, CRP, C-reactive protein
TNF-α: Tumour necrosis factor
VEGF: Vascular endothelial growth factor
TGF-β: T-cell receptor beta
ACQ: Asthma control questionnaire
AQLQ: Asthma quality of life questionnaire
HADS: Hospital anxiety depression scale
FeNO: Exhaled fraction of nitric oxide
VO_2_: Oxygen consumption
THR: Target heart rate
PEF: Peak expiratory flow
1-RM: One-maximal resistance test
FEV_1_: Forced expiratory volume in 1 second
OSA: Obstructive sleep apnoea syndrome
DLPA: Daily life physical activity
MET: Metabolic equivalent unit
WHRs: Hip circumferences and waist to hip ratios
CPET: Cardiopulmonary exercise test
SpO_2_: Oxygen saturation
WR: Work rate
VO_2_, VE: Minute ventilation
VCO_2_: Carbon dioxide production
RER: Respiratory exchange rate
HR: Heart rate
CI: Inspiratory capacity
TLC: Total lung capacity
ERV: Expiratory reserve volume, FRC, functional residual capacity
RV: Residual volume
FVC: Forced vital capacity
FEF 25-75%: Forced expiratory flow 25–75
MVV: Maximal voluntary ventilation
